# Thrombin–Fibrinogen In Vitro Flow Model of Thrombus Growth in Cerebral Aneurysms

**DOI:** 10.1055/s-0041-1728790

**Published:** 2021-05-12

**Authors:** Malebogo N. Ngoepe, Etheresia Pretorius, Ilunga J. Tshimanga, Zahra Shaikh, Yiannis Ventikos, Wei Hua Ho

**Affiliations:** 1 Department of Mechanical Engineering, University of Cape Town, Cape Town, South Africa; 2 Stellenbosch Institute for Advanced Study, Wallenberg Research Centre, Stellenbosch University, Stellenbosch, South Africa; 3 Department of Physiological Sciences, Stellenbosch University, Stellenbosch, South Africa; 4 Department of Mechanical Engineering, University of South Africa, Johannesburg, South Africa; 5 Department of Mechanical Engineering, University College London, London, United Kingdom; 6 School of Mechanical, Industrial and Aeronautical Engineering, University of the Witwatersrand, Johannesburg, South Africa

**Keywords:** cerebral aneurysms, thrombosis, in vitro, thrombin–fibrinogen

## Abstract

Cerebral aneurysms are balloon-like structures that develop on weakened areas of cerebral artery walls, with a significant risk of rupture. Thrombi formation is closely associated with cerebral aneurysms and has been observed both before and after intervention, leading to a wide variability of outcomes in patients with the condition. The attempt to manage the outcomes has led to the development of various computational models of cerebral aneurysm thrombosis. In the current study, we developed a simplified thrombin–fibrinogen flow system, based on commercially available purified human-derived plasma proteins, which enables thrombus growth and tracking in an idealized cerebral aneurysm geometry. A three-dimensional printed geometry of an idealized cerebral aneurysm and parent vessel configuration was developed. An unexpected outcome was that this phantom-based flow model allowed us to track clot growth over a period of time, by using optical imaging to record the progression of the growing clot into the flow field. Image processing techniques were subsequently used to extract important quantitative metrics from the imaging dataset, such as end point intracranial thrombus volume. The model clearly demonstrates that clot formation, in cerebral aneurysms, is a complex interplay between mechanics and biochemistry. This system is beneficial for verifying computational models of cerebral aneurysm thrombosis, particularly those focusing on initial angiographic occlusion outcomes, and will also assist manufacturers in optimizing interventional device designs.

## Introduction

Thrombosis and thrombi formation are closely associated with cerebral aneurysms, which are balloon-like structures on vessels of the brain resulting from a weakening of the vessel wall layers. 1 2 3 Aneurysm risk can be assessed through image-based screening on a population basis, of high-risk populations, clinical populations, or registries of patients. Thrombi have been observed both before and after intervention, leading to a wide variability of outcomes in patients with cerebral aneurysms. The main conundrum is that thrombi can be beneficial or destructive in unruptured cerebral aneurysms, depending on the type of clot formed. 4 5 6 7 8 9 10 The current hypothesis is that clots which partially occlude the aneurysm sac tend to lead to further vascular wall degradation, with increased risk of rupture, while those that fill the aneurysm sac contribute to stability. 3

In an attempt to harness the benefits of constructive clotting, endovascular treatment of cerebral aneurysms was designed to aid complete occlusion of the aneurysm sac. This was achieved by placement of a high surface area device in the aneurysm sac (through endovascular coiling) or by redirecting flow to the parent vessel and out of the aneurysm sac (using flow diversion devices), 11 12 always with the aim to encourage development of a stable thrombus that completely occludes the aneurysm. In the case of the latter, occlusion is not always immediate and may be observed over a period of approximately 12 months. The type of clot that forms in the aneurysm sac depends on several variables, including the form of the aneurysm, the hematological/clotting profile of the individual, and the administration of drugs during or after intervention. As a result, the process is highly patient- and protocol-specific, and a solution that works for one individual may not necessarily be suitable across the board.

The broad variability of patient outcomes has led to the development of various methodological approaches, in an attempt to better understand thrombosis in cerebral aneurysms. Clinical cases and reports have given insight into in vivo development of cerebral aneurysm thrombosis in humans, and have focused on both device-induced and spontaneous clots. 2 3 9 In vivo animal models have given insight into some of the key biological events that occur during clot formation. 13 14 15 16 17 In vitro studies aim to understand some of the general features of aneurysm thrombosis, which would otherwise be difficult to grasp in a pathology which has such wide variability among different patients. 18 19

The attempt to manage the wide variability of outcomes has led to the development of various computational models of cerebral aneurysm thrombosis. 20 21 22 23 24 25 26 The intended goal of these models is interventional planning, where prediction of outcomes such as clotting can provide additional information to the clinician about a specific patient's prospects. The current generation of models is patient specific insofar as the geometry is concerned. The use of patient-derived geometries, obtained from computed tomography and magnetic resonance imaging scans, enables calculation of flow fields that are unique to that patient. 27 The boundary conditions are not always unique, hence some amount of error is present in simulations. The clotting calculations are typically based on a single “physiological” individual's parameters, which are derived from literature obtained largely from the biochemistry community. The main challenge with several computational models is the verification and validation of predicted clotting outcomes.

To address this shortcoming, various steps have been taken toward validating in silico models. The data for validation are usually obtained from in vitro studies designed to understand the general features of cerebral aneurysm thrombosis. Ou et al presented a computational model focusing on fibrin accumulation, which is validated by data from fibrin concentration measurements in the right common carotid artery of a rat model. 24 Sarrami-Foroushani et al made use of Gester et al's model to validate their computational models, where platelets play a key role in thrombosis outcome. 18 25 Tsuji et al compared their computational model of coil embolization with in vivo clinical data. 26

Even though significant progress has been made toward validation of computational models, the main limitation with most of the in vitro experiments, on which results are based, has been the use of nonhuman tissue. The challenge of obtaining sufficient quantities of human blood to run macroscale flow experiments, over a sufficiently long time period, has meant that the most viable alternative is the use of porcine tissue and models. 13 14 18 While many studies have confirmed that porcine models demonstrate the key features of human aneurysmal disease generally, it has been shown that there are incompatibilities between human and porcine coagulation. 15 17 28 29 30 31 Some of the main differences include initiation, propagation and lysis of clots, and relative contributions of constituent parts to clot strength. 28 Furthermore, most of our current models of cerebral aneurysm thrombosis, which occurs under pathological conditions, are based on physiological biochemical frameworks. 32

In this study, we develop a simplified, macroscale, thrombin–fibrinogen flow system, based on commercially available, purified human-derived plasma proteins, which enables thrombus growth in an idealized cerebral aneurysm geometry. Soluble fibrinogen (factor I) is a glycoprotein complex that is the main clotting protein in blood. It is enzymatically converted to insoluble fibrin fibers (which form the main structural building block of a clot) by thrombin. We acknowledge that whole blood from a patient would contain platelets, erythrocytes, white blood cells, and perhaps, a plethora of circulating inflammatory biomarkers (e.g., cytokines); however, obtaining sufficient quantities of human whole blood for a macroscale experiment would be unfeasible. Our thrombin–fibrinogen model, albeit reduced, will allow us to regulate and mimic clot formation in a defined manner, without the influence of the formed blood elements, allowing us to create a well-controlled model that will generate repeatable results. In this study, we use the model to examine how mechanical and biochemical variables contribute to clot formation in an idealized cerebral aneurysm geometry. The model presented here would also be useful for validating computational models of cerebral aneurysm thrombosis or for testing endovascular device thrombogenicity, in future studies.

## Materials and Methods

This controlled experiment aimed to develop a flow phantom for a simulated cerebral aneurysm and parent vessel configuration and a host of postprocessing techniques for the data generated, by using human-derived clotting proteins (purified fibrinogen and thrombin) to recreate clot formation.

### Flow Phantom

**Fig. 1:**
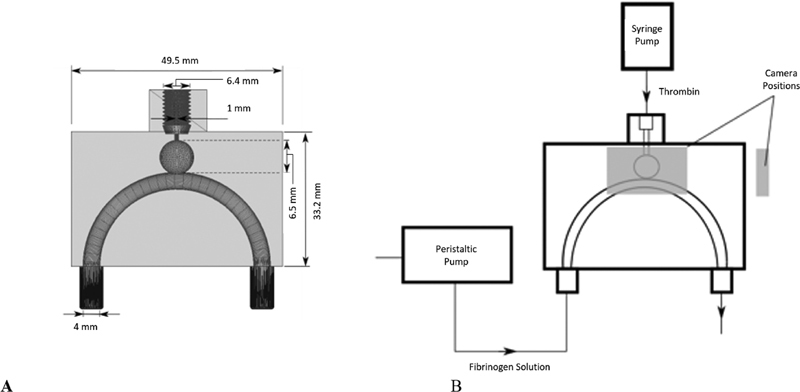
(A) Flow setup illustrating the positioning of the different components relative to the flow phantom. (B) Design of the flow phantom which includes screw top to prevent leaks.

The clotting experiment was conducted in a reusable three-dimensional (3D) printed geometry of an idealized cerebral aneurysm and parent vessel configuration, as illustrated in Fig. 1A . The idealized geometry was adapted from the work of Mulder et al. 33 The phantom was printed with a Formlabs Form 2 printer, using the standard Formlabs clear photopolymer resin. Once printed, the phantom was cleaned with isopropyl alcohol, placed in the sun, sanded, and then sprayed with a clear lacquer-based spray paint. More specific details relating to phantom preparation can be found in Ho et al. 34 The phantom included a screw mechanism at the thrombin inlet to provide a seal that prevents leaking during experimentation.

### Preparation of Clotting Factors

Human plasma-derived fibrinogen (35–65% protein) was obtained from Sigma-Aldrich (Merck Group, Missouri, United States). A fibrinogen solution of 1 mg/mL was prepared by dissolving the protein in sterile-filtered Dulbecco's phosphate buffered saline (PBS) (without calcium and magnesium). Given that the bulk of the solution comprised saline, the density was approximated at 1,000 kg/m ^3^ and the viscosity at 0.001 kg/ms. Human plasma-derived thrombin (SAE0006-150UN) was also obtained from Sigma-Aldrich and was prepared to a solution of 1 mg/mL using PBS. The prepared solutions, which were both clear, were stored in a −18°C freezer when not in use.

### Flow Setup

The flow setup is illustrated in Fig. 1B . Fibrinogen solution was pumped into the inlet of the flow phantom, using an IsmatecReglo CC pump (Ismatec, Glattbrugg, Switzerland), at different flow rates (0, 40, and 80 mL/min). Cerebral aneurysms are commonly found on blood vessels of the circle of Willis, such as the internal carotid artery, which experiences Reynolds numbers (Re) ranging from approximately Re = 200 to Re = 531. 35 A flow rate of 40 mL/min corresponds to Re = 212, while a flow rate of 80 mL/min corresponds to Re = 424, thus falling within this physiological range. Once the solution was flowing throughout the entire phantom (and all air bubbles had been removed), 10 or 100 μL of thrombin solution was injected at the top of the aneurysm sphere, using a syringe pump. The solution was not recirculated, as the fibrinogen which had been in contact with thrombin turned to fibrin, resulting in different properties. A Nikon D3300 DSLR camera (Nikon, Tokyo, Japan) was used to record the progression of the growing clot into the flow field. The experiment was repeated five times, using the same flow phantom, which was flushed out with water between experimental runs.

### Estimation of Clot Area

The clot area within the aneurysm sac was approximated using Simpleware 2020.03 (Synopsys, Exeter, United Kingdom). The photographic stills were extracted from the video recording and images were imported into Simpleware. Each image was cropped ( *x* minimum 300 mm, *x* maximum 633 mm, *y* minimum 230 mm, *y* maximum 410 mm). The image was then rescaled (scale factor 0.15) to ensure that the *x* direction matched the size of the physical phantom (49.5 mm). The image was cropped a second time ( *y* maximum 67 mm). A mask was then created and grayscale threshold values of 76 to 96 were used to select part of the clot region. Any islands greater than 200 pixels were removed and then the remainder of the clot region, not captured by the threshold, was manually selected using a paint function. Once the clot region had been selected, the surface area of the mask was calculated. This process was followed for almost all the photographs; however, some required more manual intervention than others.

### Statistical Analysis of Clot Area Results

To determine the impact of different variables on final clot growth area, two-way analysis of variance (ANOVA) for unbalanced designed was performed using MATLAB's statistics and machine learning toolbox (MathWorks, Natick, United States).

## Results

Table 1 clearly illustrates the effects of varying the fibrinogen flow rate and thrombin volume. The former alters the speed at which fibrinogen is delivered (mechanical variable), while the latter focuses on changing the amount of thrombin delivered (biochemical variable). An increase in the amount of thrombin, for a fixed flow rate of 0 mL/min, results in a drastic increase in occlusion outcome. Changes in flow rate present a more complex picture. An increase from 0 to 40 mL/min, for 10 μL of thrombin, results in an increase in occlusion percentage. An increase to 80 mL/min results in a decrease in occlusion percentage when compared with both 0 and 40 mL/min. The results for two-way ANOVA for unbalanced design, depicted in Table 2 , demonstrate that both fibrinogen flow rate and thrombin volume are significant.

**Table 1:** Occlusion percentage for experimental runs using different fibrinogen flow rates and thrombin concentrations

Case ID	Fibrinogen flow rate (mL/min)	Thrombin volume (μL)	Area (mm ^2^ )	Occlusion percentage (%)	Mean (%)	SD (%)
0_10_1	0	10	37.4	45.8	43.5	2.0
0_10_2	0	10	34.2	41.9		
0_10_3	0	10	35.0	42.8		
0_100_1	0	100	81.7	100.0	100.0	0.0
0_100_2	0	100	81.7	100.0		
0_100_3	0	100	81.7	100.0		
40_10_1	40	10	45.8	56.1	57.6	2.3
40_10_2	40	10	48	58.8		
40_10_3	40	10	49.2	60.2		
40_10_4	40	10	45.3	55.4		
80_10_1	80	10	7.1	8.7	7.7	0.9
80_10_2	80	10	5.6	6.9		
80_10_3	80	10	6.2	7.6		

Abbreviation: SD, standard deviation.

**Table 2:** Results of two-way ANOVA for unbalanced design

Source	Sum of squares	Degrees of freedom	Mean squares	F-statistic	*p* -Value
Fibrinogen flow rate	4,377.76	2	2,188.88	779.45	8.32 × 10 ^−11^
Thrombin volume	4,788.38	1	4,788.38	1,705.12	1.43 × 10 ^−11^
Fibrinogen flow rate × thrombin volume	0	0	0	0	
Error	25.27	9	2.81		
Total	13,154.03	12			

Abbreviations: ANOVA, analysis of variance; NaN, Not a Number.

**Fig. 2:**
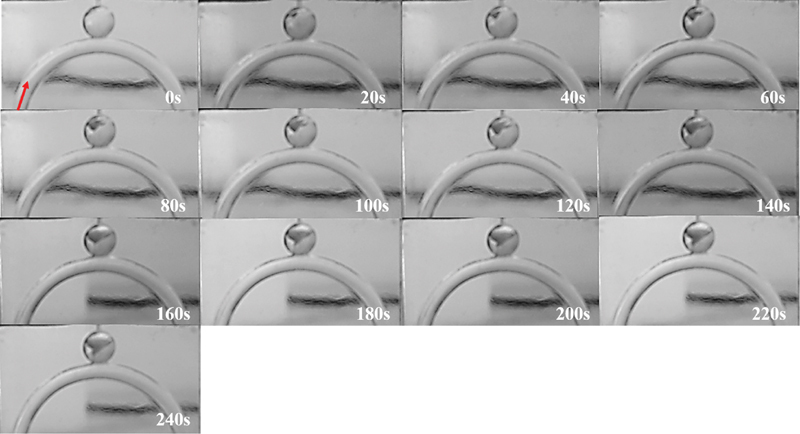
Cloth growth over time. The zero time point marks the time at which the thrombin solution is injected into the aneurysm sac and comes into contact with the flowing fibrinogen solution. The red arrow at 0 second indicates the direction of fibrinogen flow.

An unexpected, yet interesting finding is illustrated in Fig. 2 . The fibrinogen and thrombin solutions which were prepared were clear in color, resulting in the formation of a clear clot for almost every run. For the fourth 40 ml/min experimental run (40_10_4), the clot unexpectedly developed a slightly murky hue, making it possible to visualize the progression of the clot clearly, as illustrated in Fig. 2 . At 0 second, the thrombin solution is injected into the flowing fibrinogen solution. By 20 seconds, a small clot is already seen in the top left-hand corner of the sphere. As the clot grows, it propagates downward, toward the center of the sphere, and also toward the top right-hand side of the sphere. At 60 seconds, there is a clear asymmetric bias, toward the left-hand side of the sphere, where clot growth was initiated. From 80 seconds, it becomes clear that the clot front propagates toward the right-hand side, as the leftmost peak sets up a flow barrier which interrupts the circulation of flow within the sac. At the same time, the leftmost peak propagates downward (at a much slower rate than the march toward the right) and this effect is evident at ∼160 and 180 seconds. Once the clot fills a significant proportion of the upper half of the aneurysmal sac, the peak, which had migrated toward the center at 160 seconds, is seen to propagate toward the left (and downward) for the remainder of the time steps.

**Fig. 3:**
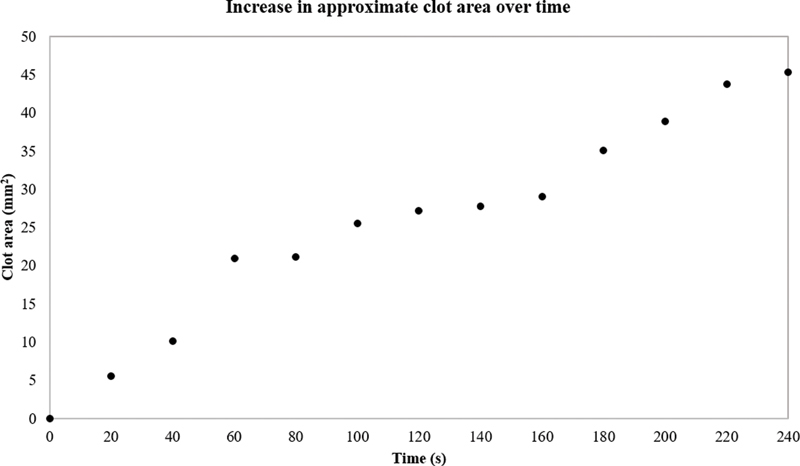
The estimated clot area from the two-dimensional images. The clot is seen to increase steadily in area over time.

Fig. 3 illustrates the approximate area of the growing clot over time for 40_10_4. The approximation does not take the 3D nature of the clot into account; however, it enables the calculation of an approximate occlusion percentage. The area of the aneurysm sac is 81.65 mm ^2^ , hence the clot area at 240 seconds (45.3 mm ^2^ ) gives an occlusion percentage of ∼55%. Even though Figs. 2 and 3 are not statistically significant, they demonstrate the type of information that could potentially be gained from this methodology. To be able to achieve such visualization for every experimental run, fluorescent fibrinogen and an appropriate laser source would be required.

## Discussion

The results from the experiment clearly demonstrate that thrombus growth in an idealized cerebral aneurysm geometry is a complex interplay between biochemical and mechanical factors. Thrombin is injected at the start of the experiment only and the clot begins as a small mass where thrombin first comes into contact with fibrinogen, resulting in the formation of an insoluble fibrin network. As more fibers are generated, the clot propagates out into regions where the flow has been slowed by the growing insoluble fiber network. In the absence of flow, diffusion is the dominant mechanism of reactant transport and the amount of thrombin in the system has a significant impact on clotting outcome. The introduction of flow, as observed in the 40 mL/min case, assists with transport of thrombin within the aneurysmal sac, enabling greater occlusion than in the 0 mL/min case. Interestingly, a further increase in flow rate (to 80 mL/min) does not amplify this effect, indicating that higher flow speeds are not necessarily supportive of clot development. The area where the clot is initiated has to have sufficiently slow flow for the reaction between thrombin and the soluble fibrinogen to take place and for the insoluble clot to develop into a fibrin fiber network that will not be destabilized by the flow. This suggests that the initiation of clotting is largely dominated, or influenced, by mechanical factors. As the clotted mass propagates into the aneurysmal sac, the flow is increasingly slowed and the rate of propagation is then limited by the reaction rate and availability of reactants. Convective flow can support clot development during the propagation process, but speeds that are too high limit the contact time between thrombin and fibrinogen, thus disrupting the fibrin formation process. While the use of a thrombin–fibrinogen in vitro model is a significant simplification of an otherwise very complex system, it is beneficial for obtaining an overall view of the main factors at play, particularly where both flow and biochemical reactions are present.

The balance between complexity and reductionism has long been debated in the study of biological systems. 36 Many of the discoveries that led to the present-day understanding of the hemostatic system were based on reductionist in vitro models that studied the impact of individual factors. From this understanding, a more complex network of the hemostatic system could be developed. Modern computing has enabled the modeling of complex biochemical signaling and flow, and has been used to explore some of the fundamental questions that remain unanswered. 37 The obvious downside of a simplified system is that it fails to account for all the in vivo variables in a patient with an aneurysm (including platelets, erythrocytes, and white blood cells, as well as circulating inflammatory biomarkers that will bind to and make the soluble fibrinogen hypercoagulable even before it interacts with thrombin). We believe that this complex comprehensive interplay of so many biological entities cannot be accounted for outside the human body. Even though the increase in complexity has proven to be largely beneficial, particularly for the hemostatic system, where many of the gaps in understanding have already been filled, there is still a place for simpler systems, such as our proposed model. In the case of the cerebral aneurysm thrombosis, there remain many questions, including the extent to which the signaling pathways of this particular disease mirror those of physiological hemostasis. Different studies have shown that the biochemical pathways of clotting are often altered by pathological states, particularly those marked by inflammation, such as cerebral aneurysms. 38 39 40 As stands, the best in vivo data that we have is percentage of occlusion at the end of endovascular device placement, as reported in clinical studies. 3 We have no data relating to the composition of the clot that forms at the end of such an intervention. As such, creating a more sophisticated in vitro model, which incorporates more blood cells, may be misguided as we would include blood cells based on physiological models of clotting. The easiest solution would be to use blood from cerebral aneurysm patients; however, we would not be able to obtain sufficient quantities for a macroscale model. The model we present here therefore gives a picture of occlusion outcome but would need to be used in conjunction with other modalities for an accurate picture of clot formation. As with other reductionist models, the greatest relevance might be in testing under very specific conditions or answering particular questions that contribute to a bigger picture. 36 41

The longer term goal of our work is to develop a virtual interventional planning tool that can give an indication of clotting outcome, based on proposed interventions, on a patient-specific, image-informed basis. The main question we wish to answer, therefore, is whether or not the placement of a device (e.g., flow diverter) results in the formation of a clot. 32 Of equal importance is whether or not the clot fills the aneurysm sac completely. The in vitro method developed here provides a technique that will enable validation of computational models developed to this end and will also enable simplified in vitro study of human clotting in flow, especially within resource constrained environments. In vivo observations of clotting in humans can be performed in the period immediately after device placement; however, it can prove that it is difficult to identify individual components and interactions within the system. Also, follow-up imaging (3, 6, and 9 months), that can be used to track development of occlusion (and inform anticoagulant pharmaceutical regimes), may be prohibitively expensive. The use of animal models has been beneficial for elucidating many of the key features of aneurysm evolution; however, pigs (and porcine models), which develop similar aneurysm characteristics to humans, are very expensive (in the region of $10,000 for recovery trials per animal). 29 The method developed here is based on a human-derived thrombin–fibrinogen model, which comprises parts that are easy to procure, and is relatively easy to implement and control. An improvement on the presented experiment would be the use of fluorescent fibrinogen and a laser source, which would ensure that the progression can be visualized for every experimental run.

Aside from the simplicity of the model, one of the main shortcomings of the method presented in this study is the lack of accurate 3D image acquisition and subsequent quantification, at this stage. The final outcome of the experiment resembles that seen in clinical practice (occlusion percentage). 12 While this is ideal for matching outcomes between virtual predictions and clinical workflows, quantification of the flow field or biochemical concentrations would be much more beneficial for model validation. It would also give greater insight into the exact contributions of different systems during clot formation. The other limitation is the time period observed during experimentation. The period defined as “immediately after endovascular device placement” is not well defined in the literature. It is therefore difficult to predict how long it would take for a clot to form immediately after intervention. On the other hand, the simplicity of the phantom and overall setup would allow the implantation of flow diverters in this setup, and thus compare directly the effect of such devices both with the preinterventional thrombus evolution and with computational models that can incorporate virtual device implantation. The rate of clot development would be influenced by, among other things, flow rate and protein concentration. Given that the work presented here is a proof of concept, optimization of these variables was not a goal at this stage, and a considerable amount of work has already gone into guidelines relating to in vitro clot experiments. 42 Rather, our aim was to determine whether or not we could grow a clot successfully, using a human thrombin–fibrinogen model in a simplified system. We also wanted to see how well we could predict occlusion outcome based on data from the experiment. This system is therefore beneficial for estimating initial angiographic occlusion and could also assist device manufacturers in optimizing designs. The model would not be suitable for studying longer term clot evolution and maturation following flow diverter placement, as more cells (e.g., platelets, erythrocytes, white blood cells, and even circulating inflammatory biomarkers such as cytokines) would be required to adequately represent the complexity of that process, particularly if we want to investigate the pathophysiological changes that occur in cerebral aneurysms.

The in vitro model presented in this article creates links between mechanics, biochemistry, and clinical outcome, using a relatively low-cost system based on human-derived proteins. The model clearly demonstrates that clot formation in cerebral aneurysms is a complex interplay between mechanical and biochemical factors. The model would be of use to device manufacturers carrying out tests of device thrombogenicity and for computational modelers seeking to validate aspects of their computational models, within the cerebral aneurysm community.

## Notes

**What Is Known on the Topic?**

- The extent to which a cerebral aneurysm is occluded by a thrombus has an impact on aneurysm outcome.
- Full occlusion is preferable to partial occlusion.
- Placement of endovascular devices for treatment of cerebral aneurysms results in occlusion of the sac.
- Computational models of cerebral aneurysm thrombosis have been developed to predict occlusion outcome.
- Validation of these computational models has been performed with animal models.

**What Does This Article Add?**

- A simplified, low-cost, in vitro method, based on human-derived clotting proteins, for predicting occlusion of cerebral aneurysms.
- Prediction of occlusion outcome, similar to clinical outcomes currently used.
- Data which can be used to validate computational models.
